# The Prognostic Value of Tumor-Infiltrating Lymphocytes in Breast Cancer: A Systematic Review and Meta-Analysis

**DOI:** 10.1371/journal.pone.0152500

**Published:** 2016-04-13

**Authors:** Yan Mao, Qing Qu, Xiaosong Chen, Ou Huang, Jiayi Wu, Kunwei Shen

**Affiliations:** 1 Breast Health Center, The Affiliated Hospital of Qingdao University, Qingdao, Shandong, China; 2 Comprehensive Breast Health Center, Ruijin Hospital, Shanghai Jiao Tong University School of Medicine, Shanghai, China; 3 Department of Oncology, Ruijin Hospital, Shanghai Jiao Tong University School of Medicine, Shanghai, China; Fondazione IRCCS Istituto Nazionale dei Tumori, ITALY

## Abstract

**Background:**

The prognostic values of tumor-infiltrating lymphocytes (TILs) and TILs subsets in breast cancer (BC) are uncertain.

**Methods:**

A systematic literature search (MEDLINE, Web of Science, EMBASE, and the Cochrane Library to August 2014) was conducted for studies which met the eligibility criteria. The primary clinical outcome was defined as disease-free survival (DFS), overall survival (OS), and BC-specific survival (BCSS). Random or fixed-effects model was adopted to estimate the summary hazard ratio (HR).

**Results:**

Twenty-five published studies comprising 22,964 patients were reviewed. Pooled analysis indicated that TILs were not prognostic markers for DFS and OS in overall population, but related to improved DFS (HR, 0.82; 95% CI, 0.76–0.88) and OS (HR, 0.79; 95% CI, 0.71–0.87) in triple negative breast cancer (TNBC) patients. For TILs subsets, CD8+ lymphocytes were associated with improved DFS (HR, 0.69; 95% CI, 0.56–0.84) and BCSS (HR, 0.78; 95% CI, 0.71–0.86) in overall population, while FOXP3+ lymphocytes were associated with reduced DFS (HR, 1.47; 95% CI, 1.01–2.05) and OS (HR, 1.50; 95% CI, 1.15–1.97). In estrogen receptor (ER) negative patients, CD8+ lymphocytes was also related to better BCSS. In addition, the high density of CD20+, CD3+ or low level of PD-1+ or γδ T lymphocytes indicated increased OS in limited studies.

**Conclusion:**

TILs and TILs subsets are promising prognostic biomarkers in breast cancer, especially in TNBC.

## Introduction

Breast cancer (BC) is the most common malignancies in women worldwide, and one of the leading causes of cancer death [1]. In BC, the bulk of evidence showed that immune cells infiltration presented in tumor, especially tumor-infiltrating lymphocytes (TILs), were associated with clinical outcomes in some malignant tumors [2–5].TILs include T cells (~75%), B cells, and natural killer (NK) cells [6], which could interrupt the immune balance during cancer development and progression. Controversies exist on how these cells present in tumor. The most convincing and reasonable hypothesis is that tumor could recruit immunosuppressive inflammatory cells to intratumoral or adjacent stromal site, and different immune cells recruited play different roles in various cancers. Since breast cancer is a complex disease with high heterogeneity, molecular subtypes including Luminal A, Luminal B, HER2 positive and triple negative breast cancer(TNBC) identified by gene expression profile or immunohistochemical panel are widely used in clinical practice, and each subtype has discrete prognostic pattern and treatment response, plenty of TILs related studies showed conflicting results in breast cancer field, the prognostic value of TILs and/or TILs subsets was not yet determined. Therefore, our meta-analysis was conducted to identify the prognostic value of TILs and/or TILs subsets in BC patient stratified by infiltration sites.

## Methods

The protocol of this study was conducted and reported in the PRISMA List (S1 Appendix).

### Search strategy

A systematic literature search was conducted within MEDLINE, Web of Science, EMBASE, and the Cochrane Library databases for original articles which met our inclusion criteria before August 2014 by using the following key words: breast cancer, lymphocytes, tumor-infiltrating, prognosis, and survival. Abstracts from the American Society of Clinical Oncology meeting and San Antonio Breast Cancer Symposium were also searched, and review articles were scanned for additional eligible studies.

### Inclusion criteria

Studies were eligible for inclusion if the following predefined criteria were met: (1) human subjects; (2) paper written in English, (3) published as original articles; (4) reported the relationship between TILs and survival outcomes in adjuvant setting (OS, DFS, BCSS, or RFS); (5) investigated the prognostic value of TILs, TILs subsets, and the ratios between the TILs subsets in BC; and (6) contained the minimum information necessary to estimate the effects (i.e., hazard ratio [HR]) and a corresponding measure of uncertainty (i.e., confidence interval [CI], *P*-values, and standard errors or variance). As an additional criterion, only the report with the most complete data was included to avoid duplication in case the same population was reported somewhere else.

### Data extraction and quality assessment

The selected articles were independently assessed by two reviewers (Y.M. and Q.Q.). The key elements related to the study design were collected from each of the included studies. The third reviewer (X.S.C.) or by contacting content experts were needed until the two reviewers reached a consensus when discrepancies appeared. The quality of each study was assessed using the established form first developed and applied by McShane et al.[7] and Hayes et al.[8]. Studies with scores ≥6 were considered high quality.

### Statistical analyses

HRs and 95% CIs were extracted from each study, which estimated the ratio of the survival possibility for high vs. low density of TILs and/or TILs subsets. We directly retrieved the HR and 95% CI from the original articles when they were provided or calculated indirectly from the Kaplan-Meier curves using the methods described by Tierney et al.[9] when they were not provided. The analyses were pooled in all patients and among different subtypes, and subgroup analyses were performed according to the locations of lymphocytes infiltration (intratumoral site, stromal site, or both sites). The interstudy heterogeneity was evaluated by chi-squared test and substantial heterogeneity was defined as *P*<0.05 or *I*^*2*^>50%. Potential sources of heterogeneity were then investigated using a predefined form in some domains reported by de Graeff et al.[10].A fixed-effects model was used if HRs were found to have fine homogeneity; if not, a random-effects model was used. Sensitivity analyses were performed for all analyses that included five or more studies, whereby studies were then omitted one by one. Publication bias was evaluated using a funnel plot with the Egger and Begg’s bias tests for the analyses involving at least 10 studies. Probable publication bias was corrected using the “trim and fill” method. All statistical analyses were performed using STATA version 12.0 (Stata Corp LP, College Station, TX, USA).

## Results

### Flow of the included studies

**Fig 1:**
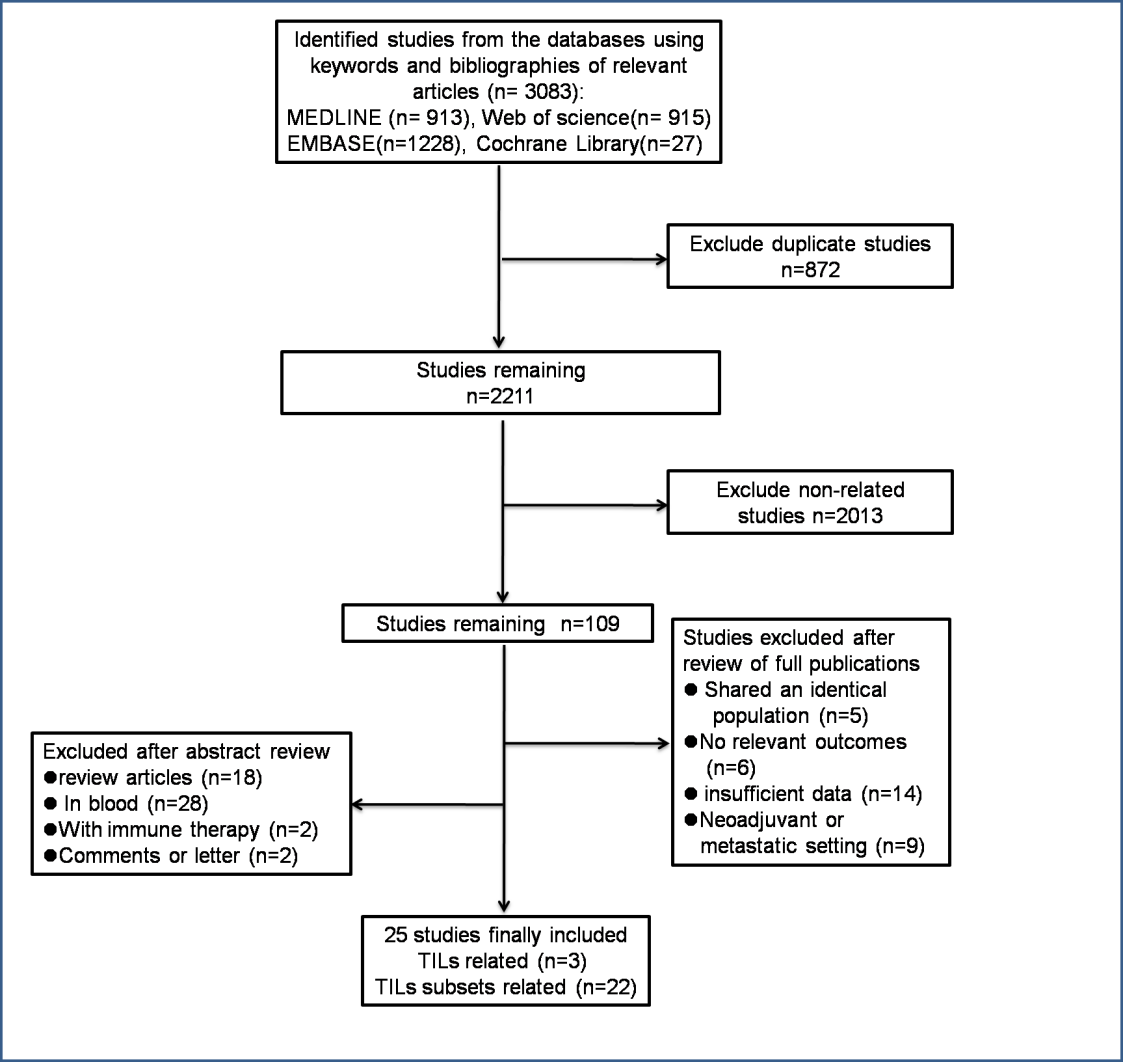
Flow chart of the studies included in the meta-analysis.

Fig 1 showed a flow chart of studies that were included in the meta-analysis. Briefly, 3,083 studies were included for initial evaluation, and 25 studies involving 22,964 patients were eligible for further assessment.

### Study characteristics

S1 Table summarized the characteristics of all included studies. All studies were published from 2006 to 2014 and conducted in Europe (n = 14), Asia (n = 10), and North America (n = 1). TILs were evaluated in 3 studies [2,11,12], and TILs subsets were evaluated in 22 studies [3,13–33]. Sample size of each study ranged from 72 to 4,520 patients, adding to a total of 22,964 patients; only 4 studies comprised less than 100 patients. The multivariate analyses of TILs and TILs subsets as prognostic factors were conducted in 23 studies. The assessment of bias for individual study presented in S2 Table showed that 22 studies were of high quality. HRs and 95% CI for overall survival (OS), BC-specific survival (BCSS), disease-free survival (DFS), or recurrence-free survival (RFS) were extracted directly from most of the studies, if available. For the few remaining studies, these were calculated using survival curves and *P* values. The most frequently used cutoff values for distinguishing the high or low density of TILs and/or TILs subsets were 10% increment (n = 3), median (n = 7), mean (n = 2), and scores calculated using several semiquantitative methods (n = 4).

### Pooled analysis of TILs

**Fig 2:**
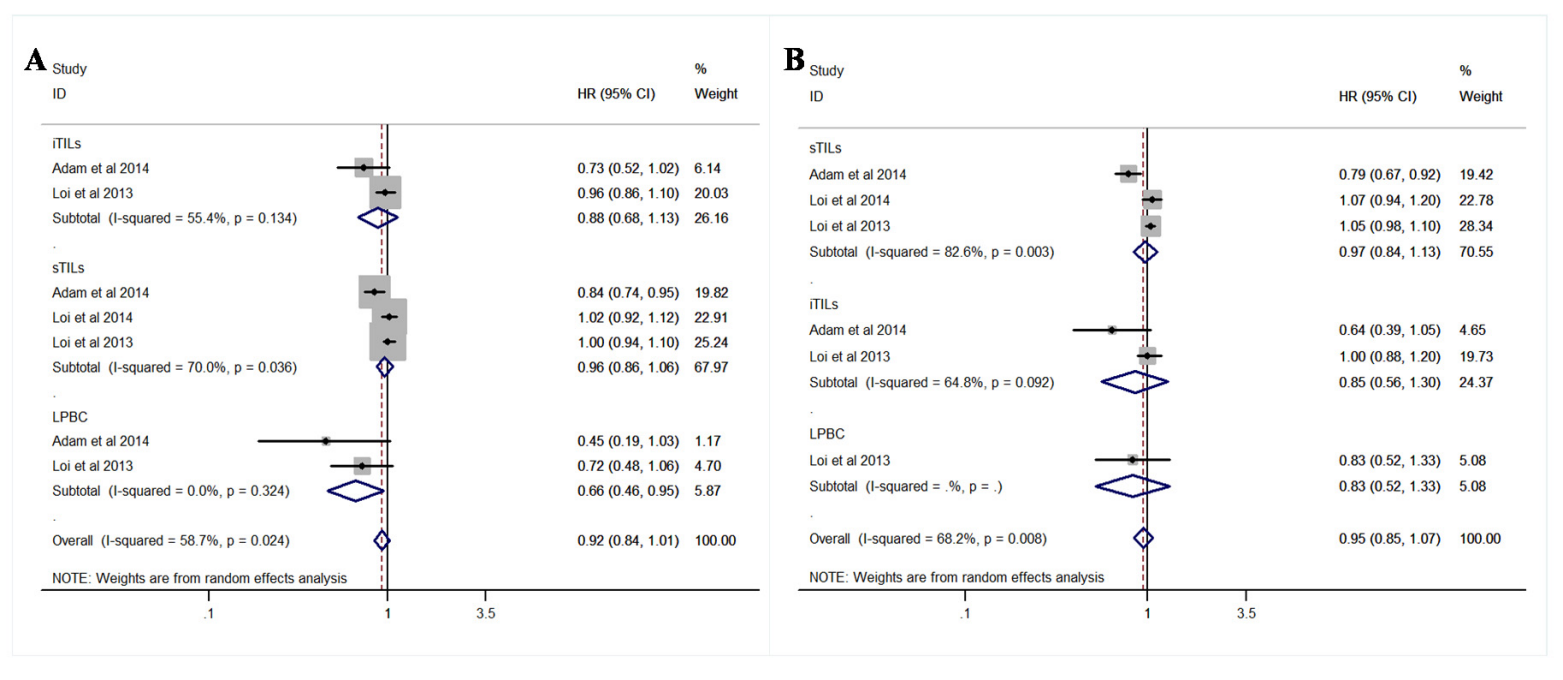
Forest plots of the random/fixed-effects meta-analysis for the efficacy of tumor-infiltrating lymphoctes for disease-free survival (DFS)(A) and overall survival (OS)(B) stratified by infiltration locations, including intratumoral site, stromal site, and both sites in breast cancer (BC) patients. The horizontal bars indicate the 95% confidence inervals (CIs)The size of the square around eacheffect estimate indicates the weight of the individual study in the meta-analysis.

Three studies [2, 11, 12] were pooled for analysis of the TILs density for DFS and OS in BC. The pooled analysis suggested that TILs were not prognostic markers for DFS (HR = 0.92; 95% CI, 0.84–1.01) or OS (HR = 0.95; 95% CI, 0.85–1.07) in overall population, but lymphocytes predominant breast cancer (LPBC), which was defined as50% infiltration of either stromal or intratumoral lymphocytic infiltration, showed a favorable impact on DFS (HR = 0.66; 95% CI, 0.46–0.95; Fig 2).

**Fig 3:**
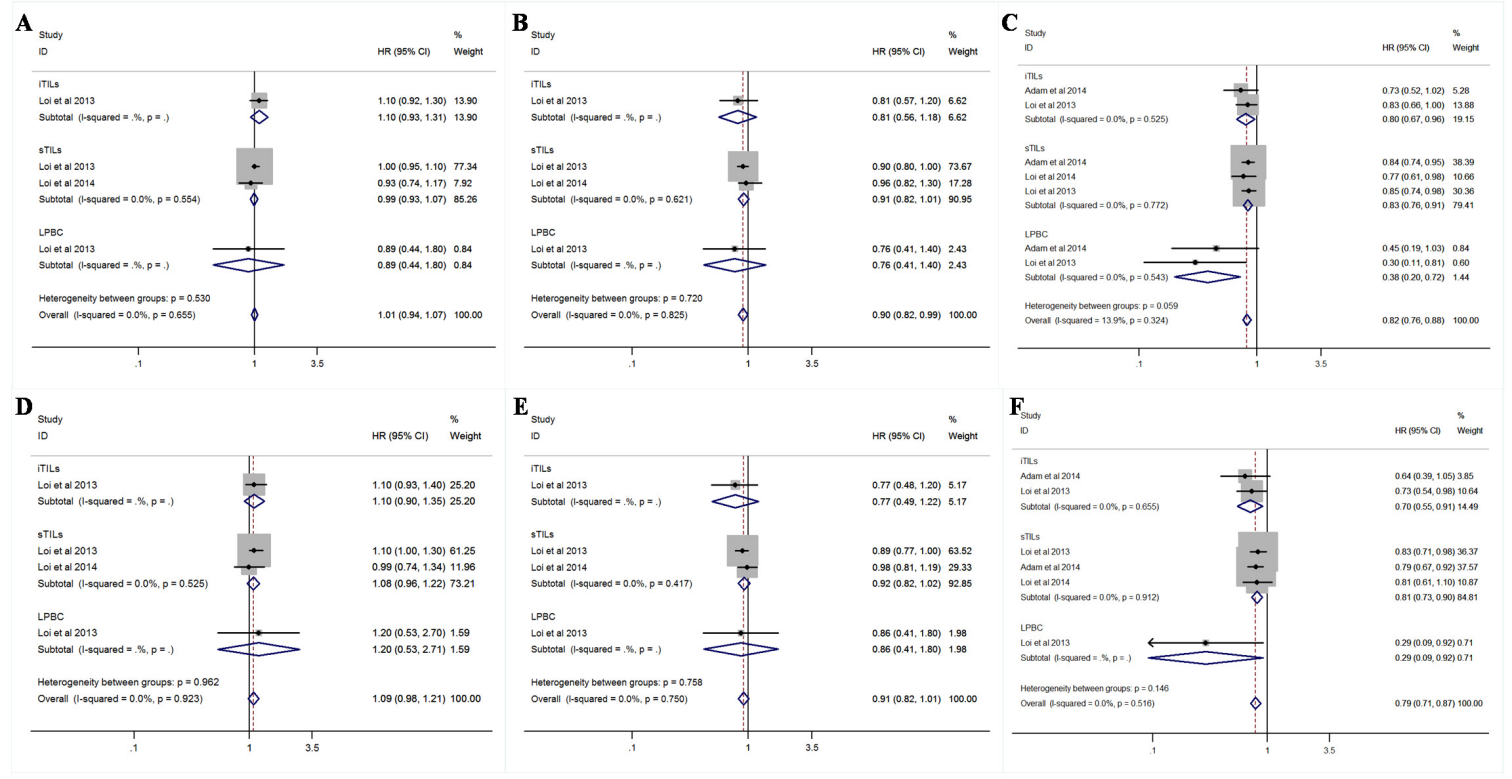
Forest plots of the random/fixed-effects meta-analysis for the efficacy of tumor-infiltrating lymphoctes for disease-free survival(DFS)(A,C,E) and overall survival (OS)(B,D,F) stratified by infiltration locations, including intratumoral site, stromal site, and both sites in ER+/HER2- (A,B), HER2+ (C,D), and triple-negative breast cancer (TNBC) (E,F) patients. The horizontal bars indicate the 95% confidence intervals (CIs). The size of the square around eacheffect estimate indicates the weight of the individual study in the meta-analysis.

As to BC subtypes, TILs also indicated survival benefit in TNBC (HR = 0.82; 95% CI, 0.76–0.88 for DFS; HR = 0.79; 95% CI, 0.71–0.87 for OS) and HER2+ patients (HR = 0.90; 95% CI, 0.82–0.99 for DFS), but not in estrogen-receptor positive (ER+) patients (HR = 1.01; 95% CI, 0.94–1.07 for DFS; HR = 1.09; 95% CI, 0.98–1.21 for OS; Fig 3). For TNBC patients, both intratumoral TILs (iTILs) and stromal TILs (sTILs) were associated with good prognosis, while LPBC indicated particularly significant survival benefit (HR = 0.38; 95% CI, 0.20–0.72 for DFS; HR = 0.29; 95% CI, 0.09–0.92 for OS; Fig 3). Sensitivity analysis (each study sequentially excluded) revealed that the result was robust and not dependent on any individual study.

### Pooled analysis of TILs subsets

Twenty-two studies [3,13–33] were assessed for the prognostic values of TILs subsets in BC patients.

#### CD8+ lymphocytes

**Fig 4:**
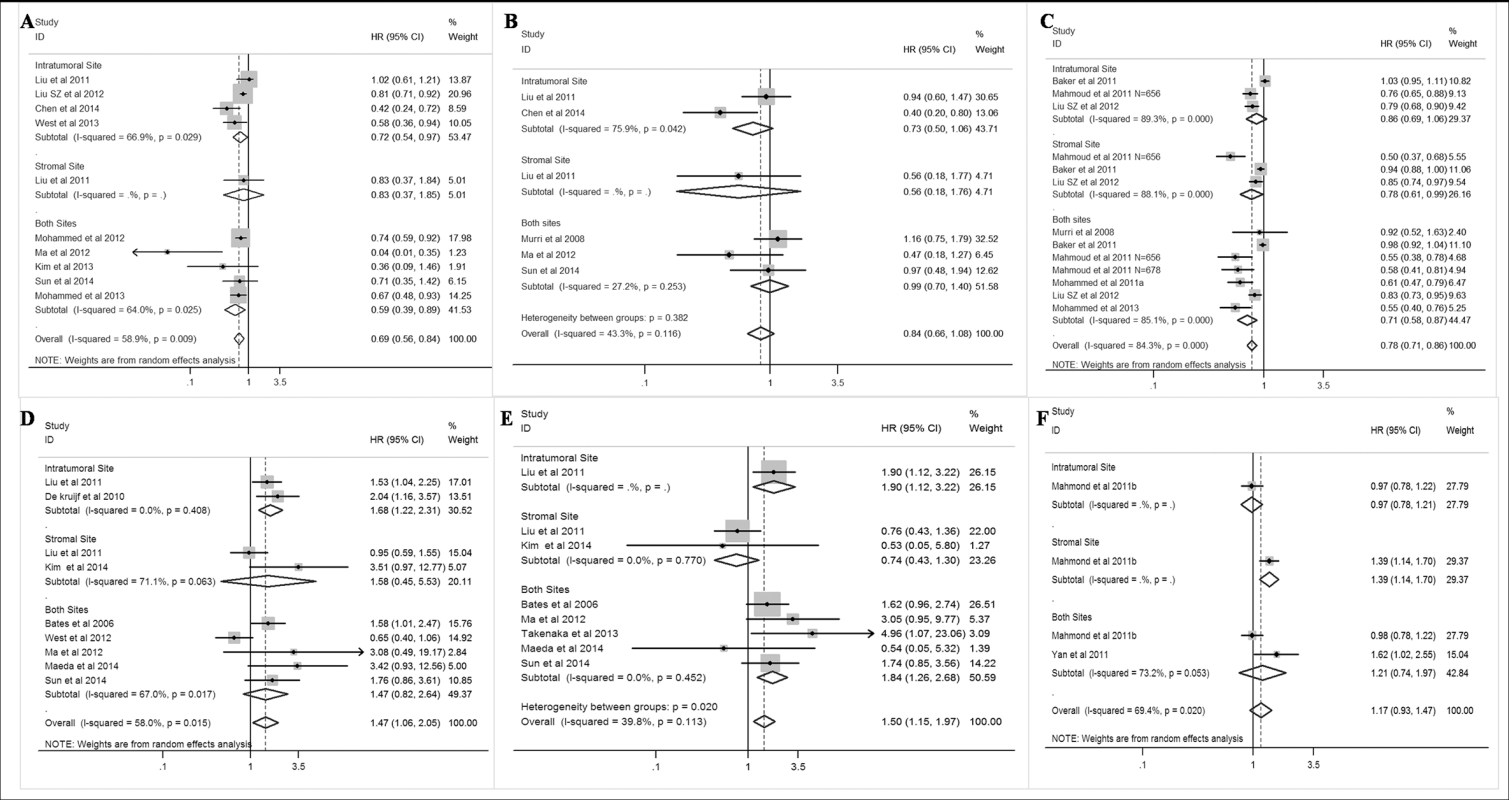
Forest plots of the random/fixed-effects meta-analysis for the efficacy of CD8+ (A,B,C) and FOXP3+ (D,E,F) lymphoctes for disease-free survival(DFS)(A,C), overall survival (OS)(B,D) and breast cancer specific survival (BCSS) stratified by infiltration locations, including intratumoral site, stromal site, and both sites in breast cancer patients. The horizontal bars indicate the 95% confidence intervals (CIs). The size of the square around eacheffect estimate indicates the weight of the individual study in the meta-analysis.

Twelve studies analyzing the prognostic value of CD8+ lymphocytes in BC patients were included in this study. The pooled analyses indicated that CD8+ lymphocytes were associated with better DFS (HR = 0.69; 95% CI, 0.56–0.84) and BCSS (HR = 0.78; 95% CI, 0.71–0.86), but not improved OS (HR = 0.78; 95% CI, 0.55–1.11; Fig 4A, 4B and 4C). Moreover, CD8+ lymphocytes infiltrated in both stromal and intratumoral sites might be a more reliable prognostic factor for DFS and BCSS. Importantly, CD8+ lymphocytes could also predict improved BCSS in ER- (HR = 0.73; 95% CI, 0.68–0.80; S1 Fig), HER2+ (HR = 0.71; 95% CI, 0.57–0.88; S2 Fig), and TNBC patients (HR = 0.64; 95% CI, 0.54–0.77; S2 Fig), but not in ER+ patients (S1 Fig). Different from ER+ patients, CD8+ lymphocytes infiltrated in any site would indicate better BCSS in ER- and TNBC patients.

#### FOXP3+ lymphocytes

In this study, FOXP3+ lymphocytes were associated with poor DFS (HR = 1.47; 95% CI, 1.06–2.05), as well as OS (HR = 1.50; 95% CI, 1.15–1.97), but not with poor BCSS (HR = 1.17; 95% CI, 0.93–1.47; Fig 4D, 4E and 4F). In subgroup analysis, FOXP3+ lymphocytes in intratumoral site might be related to reduced survival outcome. FOXP3+ lymphocytes were also associated with poor OS (HR = 3.26; 95% CI, 1.51–7.04) and BCSS (HR = 1.26; 95% CI, 1.01–1.57) in ER+ patients but not in ER- patients (S3 Fig). Limited studies analyzed the prognostic value of FOXP3+ lymphocytes in HER2+ and TNBC patients. Furthermore, CD8+/FOXP3+ ratio was not correlated with DFS (HR = 0.44; 95%CI, 0.12–1.58) or OS (HR = 0.69; 95%CI, 0.41–1.16; S4A Fig) in overall population.

#### Other lymphocytes

Limited studies evaluated the prognostic impacts of other lymphocyte subsets in BC patients. The results showed that PD-1+ and γδ T lymphocytes were associated with poor OS respectively (HR = 1.60; 95%CI, 1.15–2.23 and HR = 3.34; 95%CI, 1.21–9.23), while CD3+ lymphocytes indicated better OS (HR = 0.31; 95%CI, 0.14–0.70).CD4+ lymphocytes were not prognostic markers in breast cancer (S4B Fig). Two studies [13,25] indicated that C20+ B lymphocytes correlated with better BCSS (HR = 0.77; 95%CI, 0.61–0.96) and DFS (HR = 0.72; 95%CI, 0.58–0.89; S4C Fig) in breast cancer.

## Funnel plot asymmetry, heterogeneity, and publication bias

**Fig 5:**
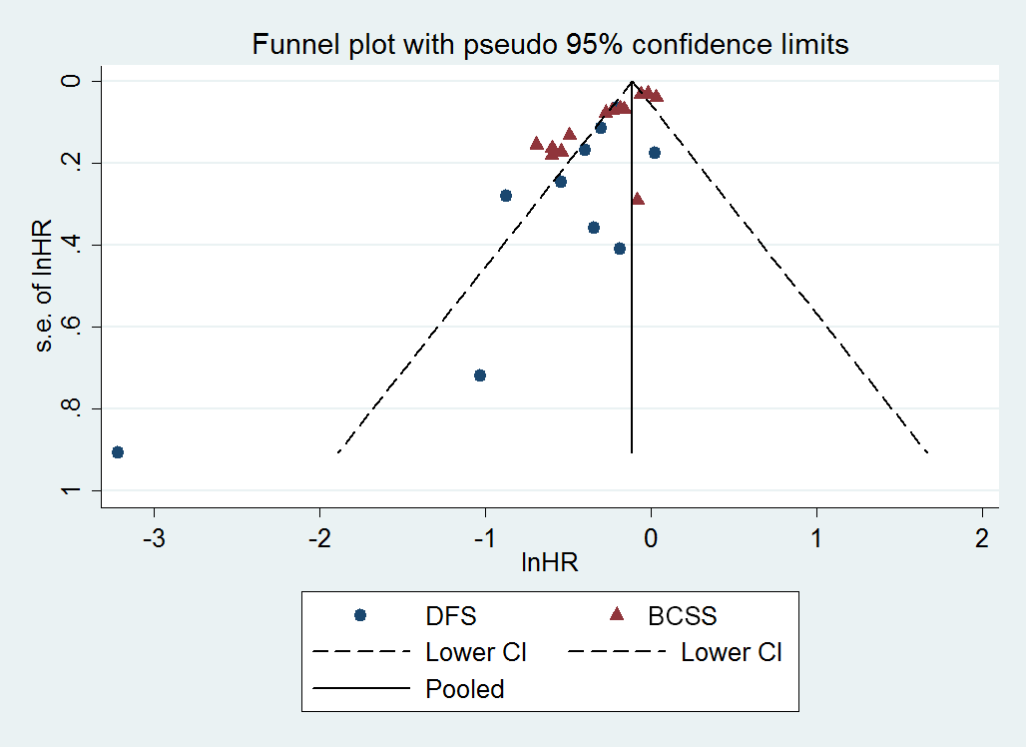
Funnel plots of the relationship between the size of the effect in individual studies and the precision of the study estimate (lnHR, horizontal axis; s.e., vertical axis) for CD8+ cells.

More than 10 studies were included to analyze prognostic value of CD8+ lymphocytes in BC patients. Begg’s and Egger’s bias tests and a visual inspection of the plots identified asymmetry in CD8+ lymphocytes (DFS and BCSS; Fig 5 and S3 Table) analysis, which indicated some bias existed and the results may not be reliable. In order to reduce the publication bias, the trim-and-fill method was conducted, 4 and 6 studies respectively were found missing in the analysis of the prognostic value of CD8+ lymphocytes in DFS and BCSS. The results suggested that effects of CD8+ lymphocytes on BCSS was changed (HR = 0.93, 95% CI, 0.84–1.03) and not reliable, while CD8+ lymphocytes still indicated better DFS (HR = 0.79, 95% CI, 0.63–0.99) after adjusted bias.

## Discussion

This meta-analysis suggested that TILs were prognostic markers for DFS and OS in TNBC patients, and also suggested good DFS in HER2+ patients, but not in overall population or ER+/HER2- patients. For TILs subsets, CD8+ and FOXP3+ lymphocytes were correlated with the prognosis of BC, but in different roles. All these results indicated that immune status of tumors in situ was very important in predicting the survival benefit in BC patients. Moreover, locations of TILs might also matter in prognosis prediction.

Since 1957, immunoediting was found in various kinds of tumor progression; however, the relationship between tumors and the immune system was complex and not fully understood. On the one hand, some types of immune cells, such as NK cells, cytotoxic T cells, and B cells, were shown to suppress the growth of cancer cells, and a larger amount of immune cells associated with better prognosis [25,34,35]. On the other hand, other types of immune cells, including macrophages and FOXP3+ Tregs, actually facilitated and promoted carcinogenesis and tumor growth [26,36]. Gu-Trantien et al.[6] found that in breast tumors in situ, the most infiltrating immune cells were T lymphocytes, which indicated that the adaptive system might play more important roles in tumor progression.

Although our meta-analysis could not prove that TILs were prognostic markers for overall breast cancer patients, TILs suggested survival benefit in TNBC patients. The results were concordant with previous studies [2,11,12]. Loi et al.[2] first found that TILs were associated with survival benefit in TNBC patients, and this result was validated by Loi et al. [12] and Adams et al. [11] respectively in FinHER and ECOG2197/1199 studies. Considering all these studies shared the same cutoff of TILs and randomized design, the prognostic value of TILs in TNBC patients who received adjuvant chemotherapy were solid and convincing. Moreover, TILs in breast cancer could predict better response to neoadjuvant chemotherapy[37,38],which also may indicate long term survival. Interestingly, no matter presented in pre-treatment biopsy or post-treatment residual tissue, TILs were consistently associated with good prognosis in TNBC patients [39,40], which might suggest that pre- and post-treatment TILs could be surrogate markers for measuring treatment efficacy in TNBC patients. For HER2+ BC, Loi et al.[3] found TILs were associated with good DFS in patients who didn’t receive trastuzumab treatment in BIG 02–98, while TILs were found associated with trastuzumab benefit in FinHER study[41]. In our meta-analysis, TILs only indicated better DFS in HER2+ patients, with different anti-HER2 therapy given. This still suggested that TILs could be prognostic markers for DFS in HER2+ patients who received chemotherapy. Due to limited studies and different treatment strategies, this result should be interpreted with caution and validated in more studies in future.

For TILs subsets, they have their own roles in breast cancer progression. CD8+ lymphocytes are the main effective cells in the immune response, which indicated better DFS in our study, but not improved OS. FOXP3+ Tregs had the potential to suppress effective T cells along the periphery by dampening the antitumor immunity elicited by CD4+, CD8+ T cells, dendritic cells, and NK cells. Recently, Bates et al.[16] found that Treg numbers were significantly higher in breast carcinomas than in the normal breast tissue and higher in invasive tumors than in ductal carcinoma in situ. In addition, a larger amount of infiltrating FOXP3+ lymphocytes were correlated with high-grade, positive lymph node, ER- and poor survival outcomes in invasive BC patients. Furthermore, Liu et al.[21] found that FOXP3+ Tregs infiltrated the adjacent stroma more than the tumor center, and FOXP3+ Tregs in the adjacent stroma indicated chemo-sensitive tumors. These evidences suggested that FOXP3+ lymphocytes infiltrating in situ tumors were complex. Our study suggested that FOXP3+ in intratumoral site predicted lower survival rates, even in the ER+ subtypes. However, in the metastatic breast cancer patients, Lee et al.[42] found that FOXP3+ lymphocytes indicated better progression free survival. In the neoadjuvant setting, two studies found FOXP3+ lymphocytes indicated poor survival outcome [43–45], while another study found they had no prognostic value [46]. This could possibly be explained by certain chemotherapy given, which might change the immune status in tumor [47]. Although our study found FOXP3+ lymphocytes may indicate better survival outcome in TNBC patients, due to limited studies included, this result should be understood with caution and need to be validated in more databases. A previous study found that the CD8+/FOXP3+ ratio in intratumoral site indicated a higher pathologic complete response rate but our analysis didn't find the ratio was related to DFS or OS in the adjuvant setting.

CD3, a general marker of T cells, was found associated with better OS in the adjuvant setting by Rathore et al.[28], while Heys et al.[48] found that CD3+ lymphocytes were not prognostic markers in the neoadjuvant setting. The results need further validation because of insufficient studies included. CD4+ lymphocytes are composed of T helper and regulatory cells; therefore, their roles are very complicated. Considering the limited data, more prospective studies are warranted to confirm their prognostic value in BC. Programmed cell death 1 (PD-1), a member of the CD28/CTLA-4 family of costimulatory receptors, might suppress antitumor immunity and is an important checkpoint in immunotherapy. In this study, PD-1+ lymphocytes indicated a lower OS rate in BC patients. PD-L1, a PD-1 ligand, was also found related to poor DFS and OS in BC patients, except in the luminal A subgroup [49]. Moreover, persistent high level of PD-1 expression on antigen-experienced CD8+ T cells leads to ‘‘CD8+ T cell exhaustion”, which is characterized by impaired effector function and persistent expression of inhibitory receptors [50]. This might explain why the CD8+/FOXP3+ ratio was not a prognostic marker. In addition, other markers for T cells, such as γδ T cells, were included in this study. Considering the limited number of studies, the meta-analysis could not be conducted.

Besides T lymphocytes, B lymphocytes also play important roles in cancer progression. Previously, CD20+ B lymphocytes were found associated with better DFS in epithelial ovarian cancer and improved survival in non-small-cell lung cancer. However, the prognostic value of B lymphocytes in BC is still controversial. Our study showed that the total number of CD20+ B cells was related to higher DFS and BCSS rates in BC patients, which indicated that B cells might also be important in BC progression as a part of the adaptive immune response at tumor sites.

However, the results from our study should still be interpreted with caution because we might have failed to identify some published and unpublished studies with negative results or with limited data that would have affected our pooled estimates. In addition, some studies included might have used low-quality methods, small populations, or a short follow up time frame. All these factors could also cause heterogeneity. We presumed that the potential sources of bias were as follows: (1) different cutoff values and tissue sources could result in bias. In our meta-analysis, the cutoff values were different among the studies, some studies used present or absent, while others used the mean, median, or quartiles and related statistics. Moreover, some studies tested TILs in tissue microarray while others not. These differences could be responsible for the variability in reaching a standard threshold of specific lymphocyte counts. Several experts are making efforts to develop a standardized method for evaluating TILs using hematoxylin and eosin–stained sections and to improve consistency and reproducibility in measuring TILs for future studies [51]; (2) in the analyses of TILs subsets, some studies used combined multiple markers or different kinds of ratios to predict the survival outcome of BC patients, such as CD8/FoxP3 and CD8/CD138 ratios. Due to limited studies included, these data must be interpreted with caution and investigated further in prospective studies; (3) not all of the HRs and 95% CIs were collected directly from the studies included in the meta-analysis; some HRs were derived from Kaplan–Meier survival curves when not directly provided in the original studies. To minimize this type of bias, attempts were made to contact the authors to obtain the original data; (4) published bias might confound the analyses. All data were collected by two independent reviewers and cross checked; a third reviewer was used in cases of disagreement between the first two. The trim-and-fill method was also used to adjust HRs; (5) most studies were retrospectively designed; additional prospective studies are needed to test our conclusions; (6) nearly all studies were multivariate analyzed to obtain precise estimates, adjusting for clinicopathological variables. There are still some studies that used only univariate analyses; (7) different treatment strategies. The patients included in this meta-analysis most received chemotherapy, but with different regimen and drugs. For HER2+ patients, some had anti-HER2 treatment while some not. Since chemotherapy and anti-HER2 therapy can improve the prognosis of breast cancer based on several clinical trials, different therapeutic strategies could affect the BCSS; (8) other factors. Because BC is a complicated disease, not only CD8+ lymphocytes the TILs, but other factors, such as genomic alterations, pathway activation, and microsatellite instability, also could affect the prognosis of BC patients. Further prospective studies are warranted to evaluate the values of TILs as prognostic markers in BC patients by using standardized cutoff values, strict follow up schemes, similar treatment strategies and multivariate analyses of clinicopathological variables of the patients, such as age, stage, genomic alterations, microsatellite instability, and other microenvironment factors.

## Conclusion

This meta-analysis suggests that TILs were prognostic markers for both DFS and OS in TNBC patients. In addition, a high density of CD8+ lymphocyte indicated good prognosis in BC patients, while FOXP3+ lymphocytes indicated poor survival outcomes. Immunotherapy could be a promising method by which to improve the prognosis of BC patients.

## Supporting Information

S1 Appendix PRIMSA Checklist. (DOC)

S1 Fig Forest plots of the random/fixed-effects meta-analysis for the efficacy of CD8+ lymphoctes for disease-free survival(DFS)(A,C), overall survival (OS)(A,C) and breast cancer specific survival (BCSS) (B,D) stratified by infiltration locations, including intratumoral site, stromal site, and both sites in ER- (A,B) and ER+ (C,D) breast cancer (BC) patients. The horizontal bars indicate the 95% confidence inervals (CIs)The size of the square around eacheffect estimate indicates the weight of the individual study in the meta-analysis.
(TIF)

S2 Fig Forest plots of the random/fixed-effects meta-analysis for the efficacy of CD8+ lymphoctes for disease-free survival(DFS) overall survival (OS) and breast cancer specific survival (BCSS) (C) stratified by infiltration locations, including intratumoral site, stromal site, and both sites in HER2+ (A) and triple negative breast cancer (TNBC) patients (B,C). The horizontal bars indicate the 95% confidence inervals (CIs)The size of the square around eacheffect estimate indicates the weight of the individual study in the meta-analysis.
(TIF)

S3 Fig Forest plots of the random/fixed-effects meta-analysis for the efficacy of FOXP3+ lymphoctes for disease-free survival(DFS) overall survival (OS) and breast cancer specific survival (BCSS) (B,C) stratified by infiltration locations, including intratumoral site, stromal site, and both sites in ER+ (A,B) and ER- breast cancer patients (C). The horizontal bars indicate the 95% confidence inervals (CIs)The size of the square around eacheffect estimate indicates the weight of the individual study in the meta-analysis.
(TIF)

S4 Fig Forest plots of the random/fixed-effects meta-analysis for the efficacy of other tumor- infiltrating lymphocyte (TIL) subsets for survival, including CD8+/FOXP3+ ratio (A),CD3+,CD4+,PD-1+, (B), and CD20+(C). The horizontal bars indicate the 95% confidence intervals (CIs). The size of the square around eacheffect estimate indicates the weight of the individual study in the meta-analysis.
(TIF)

S1 Table Baseline characteristics of included studies. (DOCX)

S2 Table Risk of bias assessment. (DOCX)

S3 Table Begg and Egger’s tests for funnel plot asymmetry for individual meta-analyses. (DOCX)
