# Supplementary figures and images for: The Prognostic Value of Tumor-Infiltrating Lymphocytes in Breast Cancer: A Systematic Review and Meta-Analysis
Source: PLoS One. 2016 Apr 13;11(4):e0152500. doi: 10.1371/journal.pone.0152500 (PMC4830515; doi:10.1371/journal.pone.0152500)

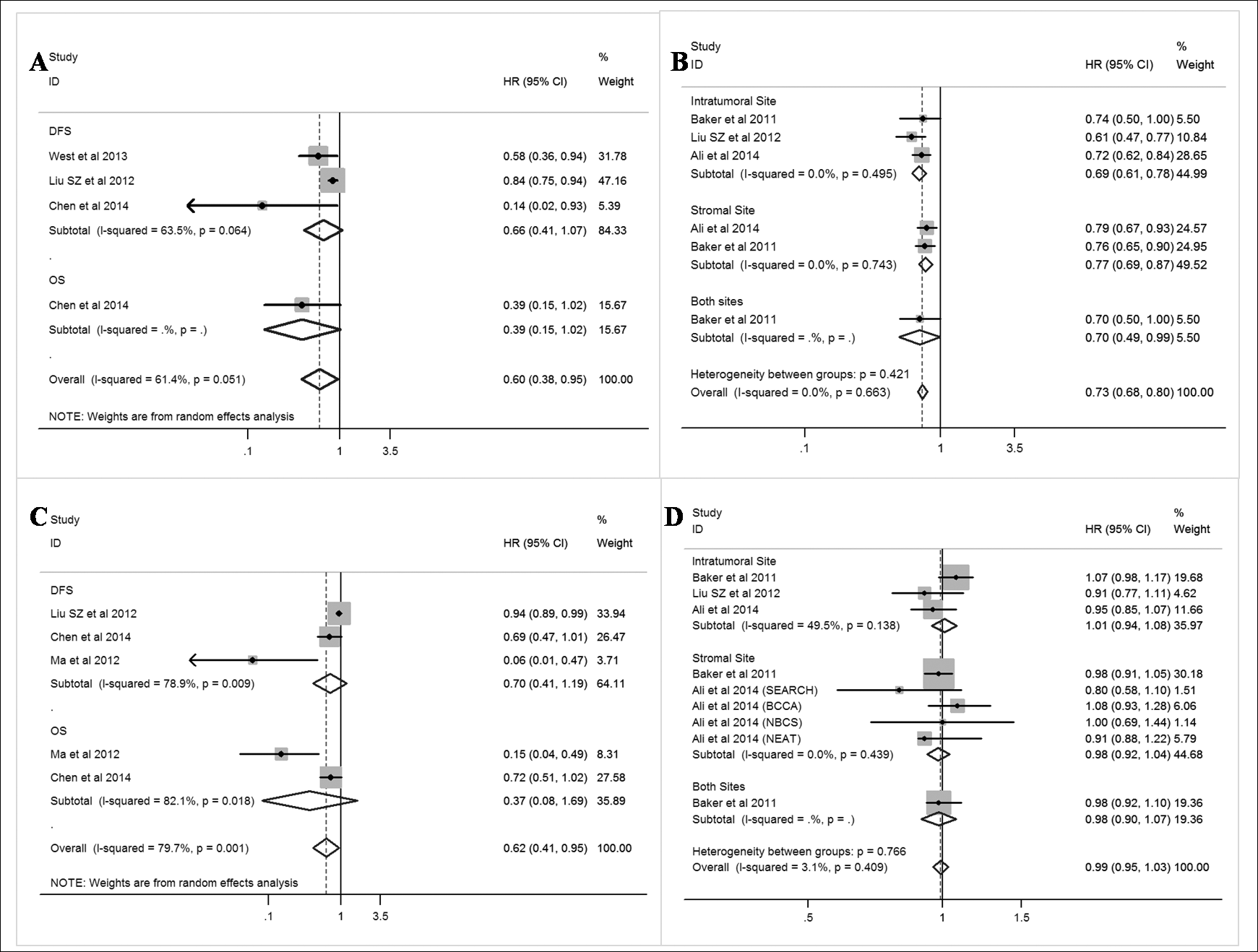

Supplement: S1 Fig — The horizontal bars indicate the 95% confidence inervals (CIs)The size of the square around eacheffect estimate indicates the weight of the individual study in the meta-analysis. (TIF) [file pone.0152500.s002.tif]

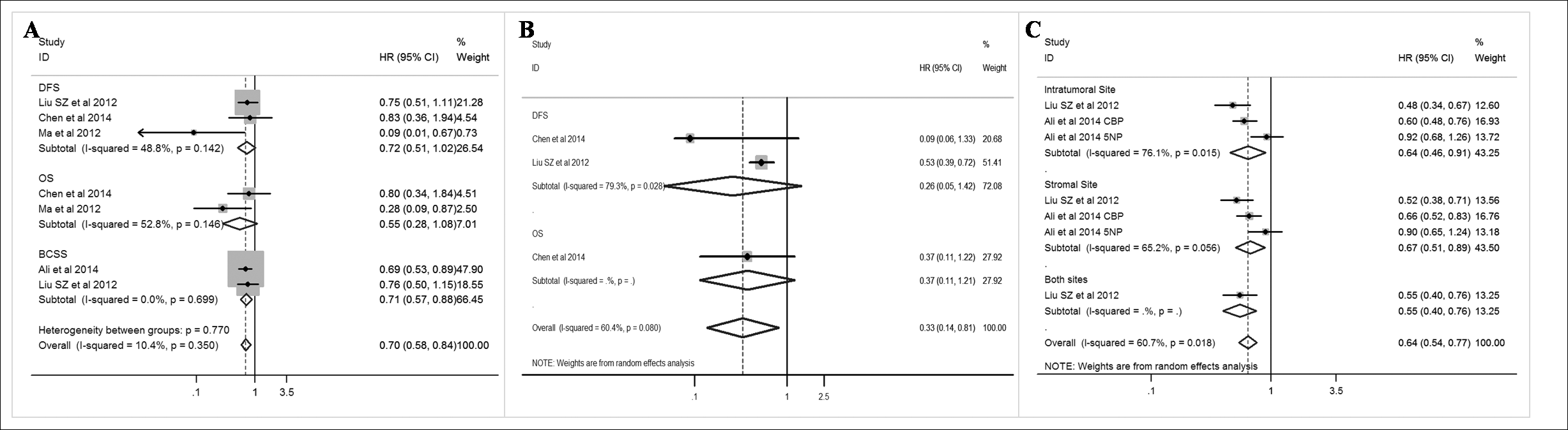

Supplement: S2 Fig — The horizontal bars indicate the 95% confidence inervals (CIs)The size of the square around eacheffect estimate indicates the weight of the individual study in the meta-analysis. (TIF) [file pone.0152500.s003.tif]

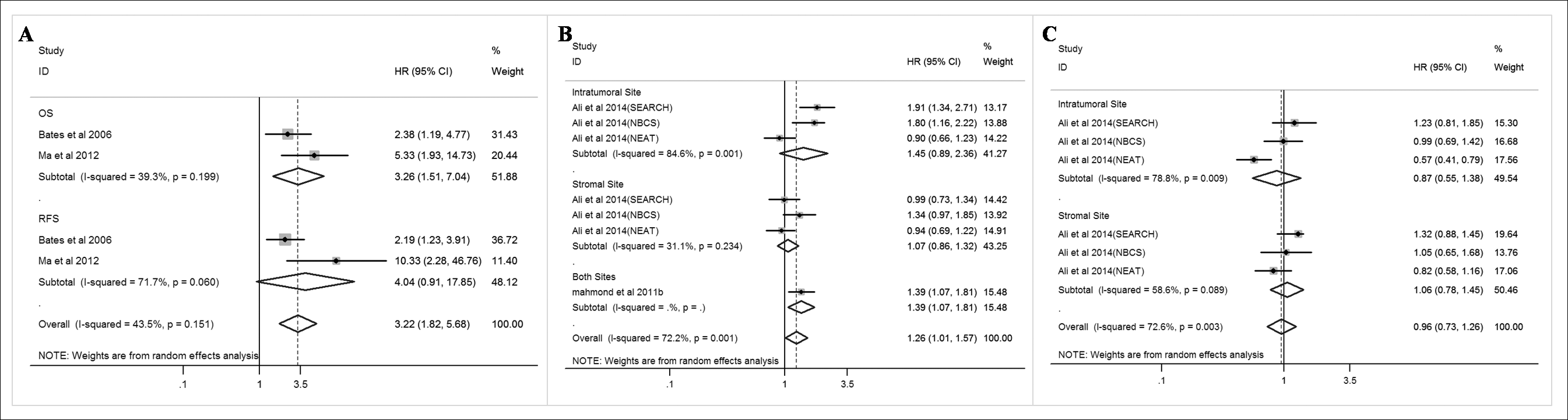

Supplement: S3 Fig — The horizontal bars indicate the 95% confidence inervals (CIs)The size of the square around eacheffect estimate indicates the weight of the individual study in the meta-analysis. (TIF) [file pone.0152500.s004.tif]

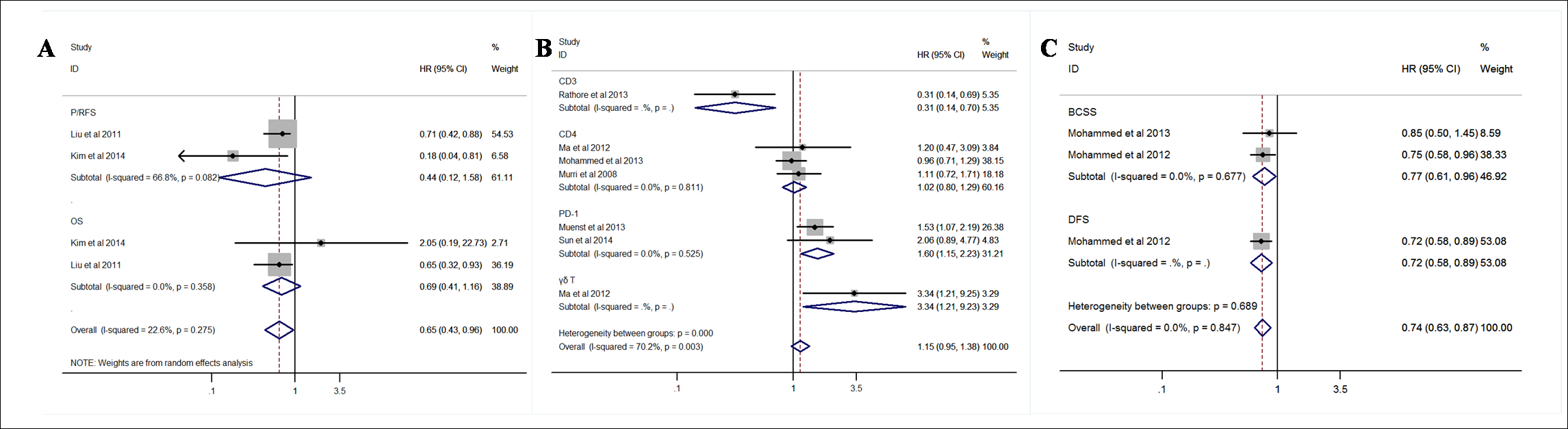

Supplement: S4 Fig — The size of the square around eacheffect estimate indicates the weight of the individual study in the meta-analysis. (TIF) [file pone.0152500.s005.tif]
