# Supplementary material for: The Prognostic Value of Tumor-Infiltrating Lymphocytes in Breast Cancer: A Systematic Review and Meta-Analysis
Source: PLoS One. 2016 Apr 13;11(4):e0152500. doi: 10.1371/journal.pone.0152500 (PMC4830515; doi:10.1371/journal.pone.0152500)
Supplement: S1 Table — (DOCX) [file pone.0152500.s006.docx]

**Table S1. Baseline Characteristics of Included Studies**

| Authors published years | Data collection | No. in study | Inclusion period | Patient | Age | Stage | Country of origin | Muti-variated factors | Marker | Assay | Cut-off points | Location | | Median follow-up time  (m) | Survival |
| --- | --- | --- | --- | --- | --- | --- | --- | --- | --- | --- | --- | --- | --- | --- | --- |
| Loi et al 2013  [2] | **Prospective** | **2009** | **1998-2001** | **LN+** | **49** | **I-III** | **Multicenter** | **-** | **TIL** | **HE** | **10% increment**  **LPBC≥50%** | **iTILs**  **Stroma**  **LPBC** | | **96m** | DFS  OS |
| Mahmoud et al 2011  [3] | **Retrospective** | **1334** | **1987-1998** | **All** | **55** | **I-III** | **UK** | **T, LN, LVI, Grade, HER2** | **CD8** | **IHC** | **≥2 cells** | **iTILs**  **adjacent,distant Stroma** | | **127**  **(4-243)** | BCSS |
| Adam  et al 2014  [11] | **Prospective** | **481** | **1998-2002** | **TNBC** | **24-85** | **I-III** | **Multicenter** | **Age, LN, T** | **TIL** | **HE** | **10% increment**  **LPBC≥50%** | **iTILs**  **Stroma**  **LPBC** | | **127.2** | OS  DFS  DFRI |
| Loi  et al 2014  [12] | **Prospective** | **935** | **2000-2003** | **All** | **50.9**  **(25-66)** | **I-III** | **Multicenter** | **Age, T, Grade, LN** | **TIL** | **HE** | **per 10% increment LPBC≥50%** | **iTILs**  **Stroma**  **LPBC** | | **62** | OS  DDFS |
| Mohammd et al 2013  [13] | **Retrospective** | **338** | **1995-1998** | **All** | **Not clear** | **I-III** | **UK** | **LVI** | **TIL**  **CD4**  **CD8**  **CD20** | **IHC** | **tertiles** | **Stroma**  **Both** | | **164** | BCSS |
| Ali  et al 2014  [14] | **Retrospective** | **12439** | **Not clear** | **All** | **Not clear** | **Not clear** | **multicenter** | **Age, T, LN, Grade, chemo, PR, HER2** | **CD8**  **FOXP3** | **IHC** | **>0** | **iTILs**  **and**  **stroma** | | **Not clear** | OS |
| Table S1. Baseline Characteristics of Included Studies (Continued) | | | | | | | | | | | | | | | |
| Baker  et al 2011  [15] | **Retrospective** | **1953** | **1985-1996** | **All** | **-** | **I-III** | **Canada** | **T , grade, LN** | **CD8** | **IHC** | **>10%** | **iTILs,stroma and**  **Both** | **63**  **(1-176)** | | BCSS |
| Bates  et al 2006  [16] | **Retrospective** | **217** | **1990-1995** | **All** | **Not clear** | **I-III** | **UK** | **T, Grade, LN, Stage** | **FOXP3** | **IHC** | **median**  **≥15** | **Both** | **87.6**  **(2.4 – 135.6)** | | OS  RFS |
| Chen  et al 2014  [17] | **Retrospective** | **332** | **1980-2001** | **All** | **-** | **I-III** | **China** | **Age, T, grade, ER, PR, HER2** | **CD8** | **IHC** | **Score>0** | **iTILs**  **Stroma** | **152** | | OS  DFS |
| De Kruijf et al 2010 [18] | **Retrospective** | **677** | **1985-1994** | **All** | **57 (23-96 )** | **I-IV** | **Japan** | **LN** | **FOXP3** | **IHC** | **>0/HPF** | **iTILs** | **228**  **(0-276)** | | RFS |
| Kim et al 2014 [19] | **Retrospective** | **143** | **2003-2007** | **All** | **Not clear** | **I-III** | **Korea** | **T, LN** | **CD3**  **CD4**  **FOXP3** | **IHC** | **median** | **iTILs**  **Stroma** | **69** | | OS  DFS |
| Kim et al 2013  [20] | **Retrospective** | **72** | **2007-2008** | **All** | **49**  **(16-83)** | **I-III** | **Korea** | **Grade, ER, PR, TNBC** | **CD4**  **CD8**  **FOXP3** | **IHC** | **mean** | **iTILs**  **and**  **adjacent stroma** | **33.7**  **(21.9-38.3)** | | DFS |
| Liu et al 2011 [21] | **Retrospective** | **1270** | **2003-2004** | **All** | **52**  **(19-92)** | **I-III** | **China** | **Age, T, LN, Grade ER,PR,HER2,chemo-,radio-, endocrine therapy** | **CD8,**  **FOXP3**  **ratio** | **IHC** | **median** | **iTILs**  **and stroma** | **66**  **(1-78)** | | OS  PFS |
| Table S1. Baseline Characteristics of Included Studies (Continued) | | | | | | | | | | | | | | | |
| Liu SZ et al 2012  [22] | **Retrospective** | **3403** | **1986-1992** | **All** | **58.9 (23-95)** | **I-III** | **British** | **Age, T, Grade, LN, LVI, subtypes** | **CD8** | **IHC** | **iTIL≥1**  **sTIL≥3**  **tTIL≥2** | **iTILs**  **stroma**  **Both** | **151.2** | | RFS  BCSS |
| Ma et al 2012  [23] | **Retrospective** | **81** | **2004-2010** | **All** | **-** | **I-III** | **America** | **Stage, LN** | **CD4,**  **CD8,**  **FOXP3** | **IHC** | **median** | **Both** | **-** | | RFS  OS |
| Maeda et al 2014  [24] | **Retrospective** | **90** | **2003-2007** | **All** | **Not clear** | **I-III** | **Japan** | **T, LN, Grade, LVI,**  **HER2,HR,B7-H3** | **FOXP3**  **B7-H3** | **IHC** | **Median** | **iTILs**  **Adjacent stroma** | **67**  **(7.8–90.5)** | | RFS |
| Mahmond et al 2012  [25] | **Retrospective** | **1470** | **1987-1998** | **All** | **55** | **I-III** | **UK** | **HER2, T, LN, grade, LVI** | **CD8 CD20** | **IHC** | **>5** | **iTILs**  **Adjacent , distant stroma** | **128 (4–247)** | | BCSS  DFS |
| Mahmond et al 2011b  [26] | **Retrospective** | **1902** | **1987-1998** | **All** | **55** | **I-III** | **UK** | **Grade, T, LN stage, vascular** | **FOXP3** | **IHC** | **intra≥1**  **stroma≥3**  **total≥3** | **iTILs**  **Adjacent , distant stroma** | **128**  **(4–243)** | | BCSS |
| Muenst  et al 2013  [27] | **Retrospective** | **660** | **1985-2007** | **All** | **64**  **(27-101)** | **I-IV** | **Switzerland** | **Age, T, LN, Grade, subtype** | **PD-1** | **IHC** | **mean** | **Both** | **65**  **(1-174)** | | OS |
| Table S1. Baseline Characteristics of Included Studies (Continued) | | | | | | | | | | | | | | | |
| Rathore et al 2013  [28] | **Retrospective** | **127** | **-** | **All** | **50**  **(25-86)** | **I-IV** | **India** | **Grade, LN, stage** | **CD3** | **IHC** | **>25/HPF** | **iTILs**  **and**  **Stroma** | **42** | | DFS |
| Sun et al 2014  [29] | **Retrospective** | **208** | **2004-2008** | **All** | **57.6**  **( 31-85)** | **I-III** | **China** | **LN, T, Grade** | **PD-1**  **CD8**  **FOXP3** | **IHC** | **median** | **iTILs**  **and**  **adjacent stroma** | **72**  **8.04–102.24**  **.** | | OS  DFS |
| Takenaka et al 2013  [30] | **Retrospective** | **98** | **1995-2005** | **All** | **-** | **I-III** | **Japan** | **Grade, ER, HER2** | **FOXP3** | **IHC** | **score≥2** | **iTILs**  **and**  **adjacent stroma** | **-** | | OS |
| West et al 2013  [31] | **Retrospective** | **175** | **1988-2000** | **ER-** | **-** | **I-III** | **Canada** | **T, LN, chemotherapy** | **FOXP3**  **CD8** | **IHC** | **>18**  **per mm^2^** | **iTILs**  **and**  **Stroma** | **83** | | RFS |
| Yan et al 2011 [32] | **Retrospective** | **491** | **-** | **All** | **55**  **(24-87)** | **I-III** | **UK Australia** | **Age, T, LN, Grade, ER,HER2,chemo-,**  **endocrine therapy** | **FOXP3** | **IHC** | **≥15 per core** | **Both** | **131.9** | | BCSS |
| Murri et al 2008  [33] | Retrospective | 168 | 2001.6-2002.12 | All | Not clear | I-III | UK | - | CD4  CD8  CD68 | IHC | median | Both | 72 | | BCSS  OS |

Abbreviations: Both=both intratumor and stroma; sTIL= tumor infiltrating lymphocytes in tumor stroma; iTIL= tumor infiltrating lymphocytes in epithelial tumor cells;
