# Supplementary material for: The Prognostic Value of Tumor-Infiltrating Lymphocytes in Breast Cancer: A Systematic Review and Meta-Analysis
Source: PLoS One. 2016 Apr 13;11(4):e0152500. doi: 10.1371/journal.pone.0152500 (PMC4830515; doi:10.1371/journal.pone.0152500)
Supplement: S2 Table — (DOCX) [file pone.0152500.s007.docx]

**Table S2. Risk of Bias Assessment**

| Study | Is the population under study defined with in- and exclusion criteria? | Were patient data prospectively collected? | Are the main prognostic patient and tumor characteristics presented?^1^ | Is the method used for determination of protein expression specified? | Is the IHC or HE staining protocol specified?^2^ | Were stainings evaluated by > 1 observer? | Is the study endpoint defined? | Is the time of follow up specified? | Is loss during analysis or follow up described? | Quality rating |
| --- | --- | --- | --- | --- | --- | --- | --- | --- | --- | --- |
| Loi et al 2013 [2] | **1** | **1** | **1** | **1** | **1** | **1** | **1** | **1** | **1** | 9 |
| Mahmoud et al  2011 [3] | **0** | **0** | **1** | **1** | **1** | **1** | **1** | **1** | **1** | 7 |
| Adam et al 2014 [11] | **1** | **1** | **1** | **1** | **1** | **1** | **1** | **1** | **1** | 9 |
| Loi et al 2014 [12] | **1** | **1** | **1** | **1** | **1** | **1** | **1** | **1** | **1** | 9 |
| Mohammed et al  2013 [13] | **0** | **0** | **1** | **1** | **1** | **1** | **1** | **1** | **0** | 6 |
| Ali et al 2014 [14] | **0** | **0** | **1** | **1** | **1** | **0** | **1** | **1** | **0** | 5 |
| Baker et al 2011 [15] | **0** | **0** | **1** | **1** | **1** | **Not clear** | **1** | **1** | **1** | 6 |
| Bates et al 2006 [16] | **0** | **0** | **1** | **1** | **1** | **0** | **1** | **1** | **1** | 6 |
| Chen et al 2014 [17] | **0** | **0** | **1** | **1** | **1** | **1** | **1** | **1** | **1** | 7 |
| De Kruijf  et al  2010 [18] | **0** | **0** | **1** | **1** | **1** | **1** | **1** | **1** | **0** | 6 |
| Kim et al 2014 [19] | **0** | **0** | **1** | **1** | **1** | **Not clear** | **1** | **1** | **0** | 5 |
| Kim et al 2013 [20] | **0** | **0** | **1** | **1** | **1** | **1** | **1** | **1** | **1** | 7 |
| Table S2. Risk of Bias Assessment (Continued) | | | | | | | | | | |
| Study | **Is the population under study defined with in- and exclusion criteria?** | **Were patient data prospectively collected?** | **Are the main prognostic patient and tumor characteristics presented?^1^** | **Is the method used for determination of protein expression specified?** | **Is the IHC or HE staining protocol specified?^2^** | **Were stainings evaluated by > 1 observer?** | **Is the study endpoint defined?** | **Is the time of follow up specified?** | **Is loss during analysis or follow up described?** | Quality rating |
| Liu et al 2011 [21] | **0** | **0** | **1** | **1** | **1** | **1** | **1** | **1** | **0** | 6 |
| Liu SZ et al 2012 [22] | **0** | **0** | **1** | **1** | **1** | **1** | **1** | **1** | **1** | 7 |
| Ma et al 2012 [23] | **0** | **0** | **1** | **1** | **1** | **1** | **1** | **1** | **1** | 7 |
| Maeda et al 2014 [24] | **0** | **0** | **1** | **1** | **1** | **1** | **1** | **1** | **0** | 6 |
| Mahmond et al  2012 [25] | **0** | **0** | **1** | **1** | **1** | **1** | **1** | **1** | **1** | 7 |
| Mahmond et al  2011 [26] | **0** | **0** | **1** | **1** | **1** | **1** | **1** | **1** | **1** | 7 |
| Muenst et al 2013 [27] | **0** | **0** | **1** | **1** | **1** | **Not clear** | **1** | **1** | **1** | 6 |
| Rathore et al 2013 [28] | **0** | **0** | **1** | **1** | **1** | **1** | **1** | **1** | **1** | 7 |
| Sun et al 2014 [29] | **0** | **0** | **1** | **1** | **1** | **1** | **1** | **1** | **0** | 6 |
| Takenaka et al  2013 [30] | **0** | **0** | **1** | **1** | **1** | **1** | **1** | **1** | **1** | 7 |
| West et al 2013 [31] | **0** | **0** | **1** | **1** | **1** | **0** | **1** | **1** | **1** | 6 |
| Yan et al  2011 [32] | **0** | **0** | **1** | **1** | **1** | **Not clear** | **1** | **1** | **0** | 5 |
| Murri et al 2008 [33] | 0 | 0 | 1 | 1 | 1 | 1 | 1 | 1 | 0 | 6 |

Abbreviations: IHC = immunohistochemistry; HE staining=Hematoxylin-eosin staining

^1^At least four of the following characteristics: age at diagnosis, stage, tumor type, differentiation grade. ^2^At least four of the following criteria: antigen retrieval, primary antibody, dilution, detection method, cut-off value for positive expression.
