# Supplementary material for: The Prognostic Value of Tumor-Infiltrating Lymphocytes in Breast Cancer: A Systematic Review and Meta-Analysis
Source: PLoS One. 2016 Apr 13;11(4):e0152500. doi: 10.1371/journal.pone.0152500 (PMC4830515; doi:10.1371/journal.pone.0152500)
Supplement: S3 Table — (DOCX) [file pone.0152500.s008.docx]

**Table S3: Begg’s and Egger’s tests for funnel plot asymmetry for individual meta-analyses**

| **Analysis** | **Begg’s Kendall’s tau** | ***Begg’s p*** | **Egger’s Regression coefficient** | ***Egger’s p*** |
| --- | --- | --- | --- | --- |
| **CD8+ and DFS** | 1.43 | *0.152* | -2.39 | ***0.044*** |
| **CD8+ and BCSS** | 2.38 | ***0.017*** | -3.71 | ***0.000*** |
